# Artificial Food Color Additives and Child Behavior

**DOI:** 10.1289/ehp.1104409

**Published:** 2012-01-01

**Authors:** Mitchell A. Cheeseman

**Affiliations:** Office of Food Additive Safety, Center for Food Safety and Applied Nutrition, Food and Drug Administration, College Park, Maryland, E-mail: mitchell.cheeseman@fda.hhs.gov

In his commentary, Weiss (2012) discusses results of the recent Food and Drug Administration (FDA) evaluation of the possible association between artificial food color additives (AFCs) and adverse behaviors in children, including those related to hyperactivity. The stated aim of the commentary is “to examine the basis of the FDA’s position, the elements of the review that led to its decision and that of the committee, and the reasons why this is an environmental issue.” In the commentary, however, *a*) the FDA’s petition review and safety assessment processes are misconstrued; *b*) the range of normal behaviors and the levels at which these behaviors can be considered adverse are not distinguished, and comparisons that cloud the distinction are unsupported; *c*) examples from individual studies are used out of context or irrespective of the conclusions expressed by the authors; *d*) specific results are cited from studies the FDA concluded were fundamentally flawed; and *e*) comprehensive reviews by other scientific panels are not mentioned. As a result, the viewpoint presented does not properly characterize the public health issue, the FDA’s evaluation and conclusions, or the processes involved, including the FDA’s proposed actions. This letter addresses as many general errors, omissions, and apparent flaws in the commentary as space permits.

In 2008, the Center for Science in the Public Interest (CSPI) petitioned the FDA to ban eight AFCs based primarily on results from clinical challenge studies on behavioral effects of these chemicals in children with a history of hyperactivity disorders or related behavioral problems (CSPI 2008). The petition also cited studies that tested potential effects of AFCs in children without behavioral problems (e.g., McCann et al. 2007) or assessed the effects of the Feingold diet, which eliminates more than just AFCs (e.g., Conners et al. 1976; Harley et al. 1978). In direct response to the petition and based on the breadth of the literature cited, the FDA assessed not only the hypothesis that AFCs trigger or exacerbate “hyperactivity” and attention deficit/hyperactivity disorder (ADHD), as noted in the commentary, but rather considered all treatment-related behavioral effects from relevant clinical studies on AFCs. This was stated in direct and unequivocal language in the FDA’s Food Advisory Committee (FAC) meeting notice in the *Federal Register* (FDA 2010): The FAC’s agenda was “to discuss whether available relevant data demonstrate a link between children’s consumption of synthetic color additives in food and adverse effects on behavior,” and that is how the committee considered the matter at the meeting.

As understood and incorporated in the FDA petition review process, confidence in the reliability of a study’s findings must be determined through scientific review using appropriate criteria before proper interpretation and applicability can be determined. Only then can results be considered in the context of all studies reviewed and a final comprehensive interpretation rendered. Using data out of context of study design and without regard to reliability and sound interpretation result in improper characterization of the issue and misdirection for future research. For example, Weiss (2012) stated that the McCann et al. (2007) study “demonstrated statistically significant adverse responses in both groups of children to the food color challenge.” Several uncertainties in that study stemming from issues and confounders related to study design and outcome measures were not mentioned, such as *a*) inclusion of a preservative (sodium benzoate) and different challenge color mixes in the two age groups of children; *b*) inconsistencies between parental observations and clinical or teacher observations; and *c*) characterization of a treatment effect as adverse when it may, in fact, fall within the normal range of childhood behavior. The evaluations of the McCann study by both the FDA and the European Food Safety Authority (EFSA 2008) considered it equivocal and of uncertain biological relevance. In the commentary, effect size is cited in support of the overinterpretation of the inconclusive results. This point ignores differences in nature and magnitude of an end point when comparing effect sizes. The examples of respiratory infection and diminished intelligence quotient (IQ) included in the commentary have narrow normal ranges; by contrast, altered behavioral activity has a much wider range, including levels of elevated activity not considered adverse, but in the range of normal activity for children.

In the commentary (Weiss 2012), there was no mention of the FDA’s conclusion that “Exposure to food and food components, including [AFC] and preservatives, may be associated with adverse behaviors, not necessarily related to hyperactivity, in certain susceptible children with ADHD and other problem behaviors, and possibly in susceptible children from the general population” (FDA FAC 2011a) is in agreement with two published meta-analysis studies, Schab and Trinh (2004) and Kavale and Forness (1983), as well as earlier conclusions of a 1982 National Institutes of Health (NIH) expert review panel (NIH 1982).

The FDA’s comprehensive literature review and weight-of-evidence analysis of the data to date support the conclusion that

Food-related triggering of problem behaviors is not due to an inherent neurotoxic property of the food or food components, including any of the artificial food colors and preservatives, but appears to result from a unique intolerance exhibited by certain predisposed children to a variety of food items and color additives. (FDA FAC 2011a)

According to Weiss (2012), this conclusion suggests that “the central nervous system is not the essential substrate for behavior or that behavior is a phenomenon independent of the brain.” The commentary is incorrect; the FDA’s conclusion is that the evidence suggests that certain food components, including AFCs, do not appear to have inherent neurotoxic properties but that some neurobiologic and/or immunologic properties of a subpopulation predispose the group to have an intolerance to specific food items, resulting in a behavioral response. These responses can vary between individuals in nature, magnitude, and triggering item. In contrast to the inference in the commentary, the FDA’s evaluation (FDA FAC 2011a) also proposed the need for research to characterize the underlying properties of this sensitivity so that any potentially vulnerable subpopulation can be clearly identified and any appropriate additional steps can be taken to ensure that the group is protected.

In his commentary, Weiss (2012) also erroneously stated that the “FDA reviewed the available evidence and concluded that it did not warrant further agency action.” The FDA has not reached any such conclusion. The FDA is reviewing recommendations made by the FAC, as well as public comments submitted in response to the meeting, including issues presented in the commentary, as we continue our review of the information and decide how to move forward on this matter.

I hope that this letter helps to clarify the FDA’s evaluation and position with regard to the possible association between AFCs and problem behaviors in children, including those related to hyperactivity. Any party interested in further clarification of the FDA’s evaluation, the CSPI petition review, and the FAC can access relevant, detailed information online from the FAC (FDA FAC 2011b).
